# Impact of COVID-19 Restrictions on Elderly Mobility and Emergency SOS Alarm Responses: A GPS-Based Study in the Czech Republic

**DOI:** 10.3390/healthcare12232442

**Published:** 2024-12-04

**Authors:** Vít Janovský, Marek Maška, Karel Hána

**Affiliations:** 1 University Centre for Energy Efficient Buildings, Czech Technical University in Prague, 273 43 Buštěhrad, Czech Republic; 2 Faculty of Biomedical Engineering, Czech Technical University in Prague, 272 01 Kladno, Czech Republic

**Keywords:** elderly SOS alarms, mobility, pandemic (COVID-19), GPS tracking

## Abstract

Background: Understanding the impact of pandemic measures on elderly mobility is crucial for shaping public health responses. This retrospective study assesses the changes in mobility of 216 elderly individuals (average age 84 years) in the Czech Republic before and during COVID-19 restrictions, using two years of GPS tracking data. Methods: We conducted statistical analyses across various physical, demographic, and social factors. Data were analyzed for mobility patterns, SOS alarm usage, and relationships between mobility and factors such as age group, body mass index, sex, city size, and housing type. Results: During the study, 4409 SOS alarms were triggered, with only 16.6% requiring action. Alarms triggered outside the home decreased from 34% pre-COVID-19 to 29% during COVID-19, reflecting reduced outdoor activity. Mobility decreased by 43.9% overall during the pandemic. The largest year-on-year decline in mobility was in May, reaching 57.6%. However, 10% of the participants exhibited increased mobility. Factors such as age group, body mass index, sex, city size, and type of housing were analyzed, and we found that city size and housing type did not significantly influence mobility levels. Conclusions: These findings highlight the substantial decline in elderly mobility during the COVID-19 pandemic and emphasize the need for tailored interventions, such as outdoor mobility programs and telemedicine, to support elderly populations during public health crises.

## 1. Introduction

The COVID-19 pandemic led to lockdowns and movement restrictions, which significantly reduced social interactions and mobility among elderly populations [1]. These measures had a particularly strong impact on the mobility of individuals, especially the elderly, whose ability to engage in community activities such as shopping, visiting libraries, and socializing at cafes or pubs was severely limited [2]. While previous studies have explored the effects of COVID-19 on elderly mobility using subjective data, this study utilizes objective GPS tracking to provide a precise evaluation of mobility changes, addressing a gap in the existing research. Formal exercises like visits to daycare centers, walks, and other outings also declined. Reduction in mobility can have detrimental health consequences, particularly for older adults, as mobility is closely linked to overall well-being, even though it is not directly equivalent to physical activity [3]. Participants with limited baseline neighborhood mobility had lower active aging scores than those with daily mobility, though both groups experienced similar decline over time. Interestingly, those with limited mobility retained certain activities, like making their day more interesting and engaging in faith or worldview pursuits, which reduced differences for this group during COVID-19 [4].

For the elderly, mobility often serves as a proxy for their functional radius of action, i.e., the range of activities they are able to perform independently. Aging naturally affects the body’s systems, leading to a decline in both organ structure and function, and reduced mobility exacerbates this deterioration [5]. Studies conducted during the pandemic found that 33% of respondents actually reported increased physical activity following the implementation of restrictions [6]. However, the majority of elderly individuals experienced a decrease in mobility, which is known to contribute to poorer physical and psychological health outcomes [7]. Understanding this dynamic is critical as the elderly are one of the most vulnerable populations. Questionary research from Isfahan city highlighted the resilience of walking and cycling among older adults (60+), despite a general decline in travel frequency due to the pandemic [8]. Active transportation modes became more crucial post-outbreak due to fewer social constraints.

Several studies have assessed the impact of COVID-19 on mobility in various countries. However, the majority of these studies relied on subjective questionnaire data [7,9,10,11]. For instance, an international online survey of 1047 respondents found that daily sitting time increased from five to eight hours during the pandemic, indicating a significant decline in overall mobility [12]. Subjective assessments are influenced by psychological factors; the restrictions imposed by the pandemic negatively impacted individuals’ moods and perceptions [13]. This correlation between reduced mobility and poorer psychological well-being underscores the importance of accurate measurement tools.

The REMOBILIZE study [14] found that older adults in restricted settings (RS) experienced significant mobility reductions, particularly women over 70 with inactivity or functional limitations, while in non-restricted settings (NRS), decline in mobility was most notable in women with comorbidities, insufficient walking time, and highly sedentary behavior. In a study from a smaller town in Poland, only 9% of older adults reported substantial changes in mobility, although travel times and frequency declined, especially for older individuals [15]. A systematic review [16] examined the travel behavior and mobility needs of older adults across various countries during the COVID-19 pandemic, revealing substantial inequalities in mobility. Pandemic restrictions disproportionately limited essential travel for vulnerable seniors, especially those with limited social support or physical impairments, creating a pattern of more pronounced mobility reductions for seniors with fewer resources or greater health needs.

A study from Korea [17] examined factors affecting older adults’ mobility during the COVID-19 pandemic. It identified that mobility was most impacted among seniors with depression, limited physical activity, and those who spent prolonged hours sitting. Key predictors of reduced mobility included age, comorbidities, and limited access to transportation, revealing that both physical and mental health factors significantly influenced mobility patterns in older adults throughout the pandemic. An international survey study examined the mobility patterns of older adults in various residential settings during the COVID-19 pandemic. The research indicated that older adults residing in urban areas tended to spend more time on leisure trips compared with their rural counterparts. In contrast, older men in urban settings engaged less in housework-related trips than those in rural areas. These findings suggest that the built environment and available infrastructure significantly influenced the daily mobility of the elderly population [18].

While previous research has addressed the risks of increased inactivity during COVID-19, there remains a gap in studies using objective tools like GPS tracking to measure mobility in real time. GPS tracking offers a reliable method for detailed assessment of mobility patterns, particularly valuable for older adults who may underreport or inaccurately recall their activity levels when completing surveys. This study aims to fill that gap by assessing the decline in mobility during the COVID-19 pandemic with the use of GPS data and identifying the key factors that influenced these changes.

## 2. Materials and Methods

### 2.1. Participants

The study participants were elderly individuals who were in receipt of social services, specifically emergency care, while living independently in their own homes. According to the study protocol, participants were required each day to use a personal tracker with GPS, WiFi, and GSM localization [19]. All data pertaining to the research participants were anonymized to ensure the confidentiality of personal information. The researchers were granted access only to the data essential for the evaluation of the research. A comprehensive list of the data obtained is provided in Appendix B. The data for this study were exported from the emergency care system in June 2021. Given that this social service operates throughout the Czech Republic, participants were selected randomly. A total of 532 clients’ data were obtained for this research.

### 2.2. GPS Data Selection and Exclusion

**Figure 1:**
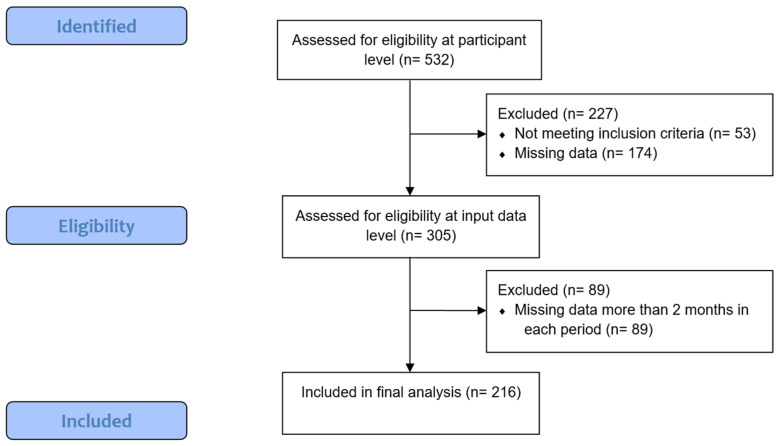
The participant selection process for the final dataset.

**Figure 2:**
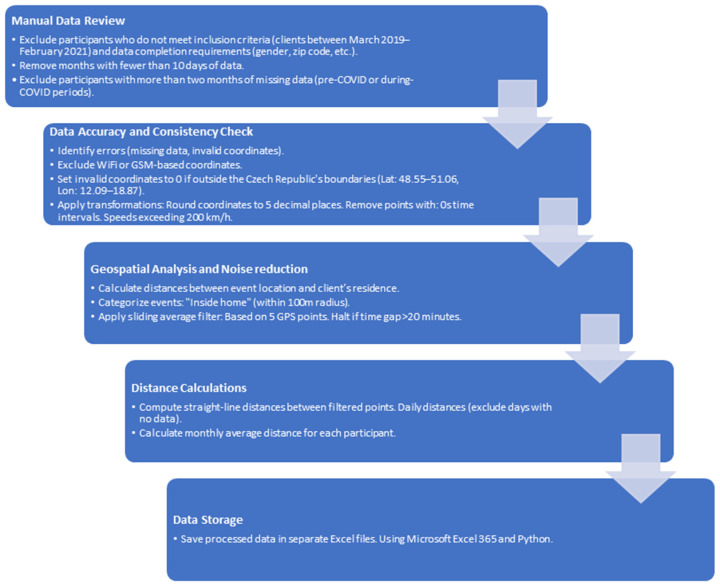
Data processing workflow.

A manual data check was conducted to ensure data quality and participant eligibility, as outlined in Figure 1. The data processing workflow is shown in Figure 2. Participants were included if they were clients of the emergency care social service between March 2019 and February 2021, with complete data including gender, zip code, and other essential details. Months with fewer than ten days of recorded data were excluded, as were participants missing data for over two months in either the pre-COVID-19 period (March 2019–February 2020) or during COVID-19 (March 2020–February 2021), resulting in 216 eligible participants.

GPS data were filtered systematically. Only true GPS coordinates (excluding WiFi or GSM-based data) were retained, rounded to five decimal places, and validated against the geographic bounds of Central Europe (latitude 48–52° N, longitude 11–18° E). Invalid points, such as those with 0s time intervals or speeds exceeding 200 km/h, were removed. A sliding average filter was applied over five points to reduce noise, halting if time gaps exceeded 20 min. Straight-line distances were calculated, daily distances were summed (excluding days with no recorded data), and monthly averages were derived for statistical analysis.

All filters and calculations were informed by investigator experience, the relevant literature (12), and manual dataset reviews, ensuring robust data for assessing participants’ mobility. Custom scripts were developed using Python, with key libraries including NumPy for numerical computations and Pandas for data manipulation.

### 2.3. Alarm Data Selection

For geospatial analysis, we calculated the distance between the event location and the client’s registered home location. Events were categorized as “inside” or “outside” the home, based on whether the event occurred within a 100 m radius around the client’s residence. This was achieved using the Haversine formula implemented via the geopy.distance.geodesic function to calculate the distance between two points on the Earth’s surface based on their latitude and longitude. Processed data were saved in Excel format for subsequent analysis, with additional steps performed using Microsoft Excel 365 and basic custom Python scripts where necessary.

### 2.4. Data Analysis

The collected data were subjected to rigorous analysis to examine the impact of COVID-19 restrictions on elderly people’s mobility. Normality testing was conducted to assess the distribution of the data. Based on the results, either a *t*-test or a Wilcoxon test was applied accordingly. Correlation tests were also performed to analyze associations between variables. Details regarding which statistical tests were used for specific data groups and hypotheses are provided in Appendix A. Statistical analysis was performed using the appropriate analytical tools and software. Descriptive statistics were used to summarize the demographic characteristics of the participants, including age, sex, and location. Mobility levels, as indicated by GPS tracker data, were analyzed to identify patterns or trends in participants’ mobility levels before and during the COVID-19 pandemic. Demographic data of the participants are shown in Table 1.

### 2.5. Governmental COVID-19 Mobility Policies

To control the spread of COVID-19, the Czech government implemented a series of escalating mobility restrictions [20,21] that had a significant impact on elderly populations. Initial measures began on 2 March 2020, when the government tightened cross-border movement, suspended flights from high-risk areas, conducted random border checks, and imposed quarantines on travelers. Public events were restricted to reduce exposure, and by March 11, all educational institutions were closed. A nationwide state of emergency was declared on March 12, which enabled the government to enforce broad movement restrictions. By March 14, non-essential businesses were ordered to close, and borders were effectively shut down on March 16.

To further protect vulnerable populations, specific shopping hours for seniors were introduced on March 18, reducing their exposure to younger individuals. Additionally, face coverings became mandatory in all public spaces on March 19, further limiting potential exposure for the elderly. These early policies, combined with quarantine and social distancing measures, aimed to safeguard older adults but contributed to a marked decline in their mobility.

In April 2020, as the initial wave appeared to stabilize, the government introduced a phased plan for lifting restrictions, allowing certain services to reopen in a limited capacity. However, the elderly remained under strict guidance to minimize outings. Throughout the summer, as cases surged again, the Czech government reinstated several restrictions, including limits on public gatherings and the reintroduction of face-covering mandates in enclosed spaces from 1 September 2020.

By October 2020, with rising infection rates, a second state of emergency was declared, bringing additional limitations on movement, especially for high-risk groups. Public gatherings were limited to six people, restaurants and cultural venues were closed, and a curfew was introduced, restricting movement between 9 p.m. and 5 a.m. Elderly individuals were strongly advised to stay at home, except for essential needs.

In late 2020 and early 2021, as cases continued to rise, the government implemented even stricter lockdown measures. In December 2020, only essential businesses remained open, and travel restrictions were tightened further. By January 2021, mandatory testing for high-risk populations and vaccination rollout plans began, initially prioritizing elderly individuals over 80. Further lockdown restrictions persisted through early 2021, with the elderly population continuing to follow stringent mobility restrictions to reduce transmission risk.

These cumulative measures—curfews, restricted shopping hours, mandatory face coverings, and quarantine mandates—were intended to protect the elderly, but they also resulted in a substantial decrease in elderly people’s daily mobility, impacting both their physical and social well-being.

### 2.6. Limitations

It is important to acknowledge the limitations of this study. The use of GPS tracker data provided objective measurements of mobility levels; however, the technology was not without errors. Erroneous data caused by reflections or other errors appeared in the GPS coordinates. Therefore, filters were deployed to remove erroneous coordinates. Furthermore, the retrospective design of the study may have introduced certain biases and limitations in data collection. These factors should be considered when interpreting the results and drawing conclusions.

### 2.7. Ethical Considerations

This research adhered to ethical guidelines and regulations concerning the protection of human subjects. The study protocol was reviewed and approved by the appropriate ethics committee, ensuring that the research was conducted in accordance with the principles outlined in the Declaration of Helsinki. Approval was granted by the Ethics Committee of Czech Technical University in Prague, Faculty of Biomedical Engineering (code C12/2021, dated 2 November 2021). The privacy and confidentiality of the participants were strictly maintained throughout the research process.

## 3. Results

### 3.1. Use of the Emergency Service

During the study period, participants triggered a total of 4409 SOS alarms (pre-COVID-19: 2090 instances; during COVID-19: 2019 instances). Of these, 4109 alarms were manually activated using SOS buttons on participants’ devices, while in 300 instances, the device detected a suspected fall. Only 732 of these alarms (405 during the pre-COVID-19 period and 327 during the COVID-19 period) required intervention from the emergency care monitoring center, representing just 16.6% of all the SOS alarms. Additionally, 11.52% (508 alarms) were categorized as test alarms. The majority, 55.3% (2438 alarms), were classified as accidental activations, highlighting a high rate of false alarms or unintentional button pressing, which indicates a potential area for improvement in future system optimization. The remaining 16.58% (731 alarms) were in the “other” category, encompassing situations not captured by the previous classifications.

With a total of 216 participants, an average of 9.67 alarms per client was recorded during the pre-COVID-19 period, and an average of 9.34 alarms per client was recorded post-March 2020. Of all the alarms, 34% were triggered outside the home during the COVID-19 period. Specifically, among alarms related to falls, 124 incidents were recorded, of which 41 were classified as serious or potentially dangerous falls. See Table 2 for a full overview.

### 3.2. Overall Decline in Mobility During COVID-19

Initially, we performed a comparative analysis of the pre-COVID-19 data (1 March 2019, to 29 February 2020) and the data from during COVID-19 (1 March 2020, to 28 February 2021) to evaluate overall mobility levels. In this analysis, we formulated three hypotheses, namely, H01, H02, and H03.

Our findings showed a significant decline in mobility among the elderly during the COVID-19 period compared with the pre-COVID-19 period. This decrease in mobility was statistically significant. Additionally, our analysis found no significant differences in mobility levels between female and male participants. Before the COVID-19 pandemic, women moved an average distance of 17.73 km per month, while men moved an average distance of 15.41 km. During the COVID-19 period, the average distance for women decreased by 44.7%, while for men it decreased by 40.9%. These observations highlight the impact of the pandemic on both genders, with a discernible reduction in daily mobility among older adults.

More analysis and statistical tests will be conducted to explore the relationship between mobility and other variables of interest, in order to provide a complete understanding of the factors influencing the decline in mobility among the elderly during the COVID-19 pandemic.

**Figure 3:**
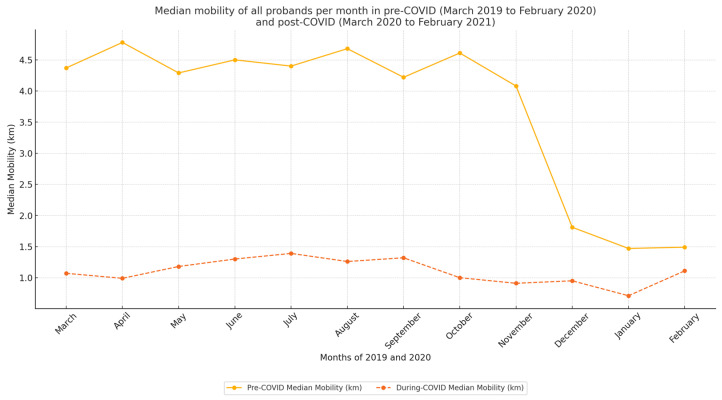
Graph displaying the mobility of all groups before and during the COVID-19 pandemic. The x axis indicates time in one-month intervals, while the y axis represents mobility in kilometers.

Table 3 summarizes the changes in mobility levels among study participants during both periods. Among the participants, 90.3% experienced a decrease in mobility levels, while 9.7% showed an improvement. Figure 3 displays plots for the full the study period, and statistical analyses for each month can be found in Table A1.

### 3.3. Monthly Trends in Elderly Mobility Before and During COVID-19

**Figure 4:**
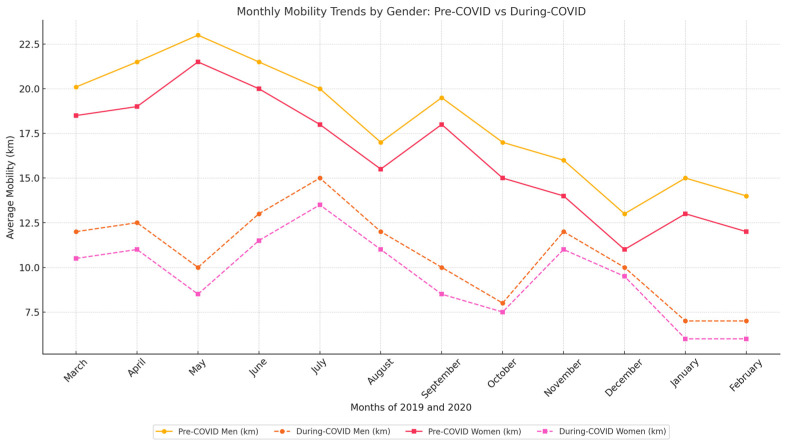
Monthly mobility trends by gender in pre-COVID-19 and COVID-19 periods. The x axis indicates time in one-month intervals, while the y axis represents mobility in kilometers.

The COVID-19 measures were maintained throughout the year, prompting our investigation to determine whether decreases in mobility were evident across individual months. To address this issue, we conducted a month-to-month comparison of data from both before and during the emergence of COVID-19. To mitigate the impact of seasonal fluctuations, such as changes in mobility associated with colder weather, we compared data from the same months in different years (e.g., January 2020 compared with January 2021). Another factor that could have influenced the results was the natural ageing of the participants, which could have led to a decline in physical fitness. In response to this, we formulated Hypothesis H04. Figure 4 shows the average monthly mobility (in kilometers) for males and females during the two observation periods: before COVID-19 and during COVID-19, with the data disaggregated by gender and plotted separately for each group.

Our analysis revealed statistically significant findings for all months. The statistical evaluation is presented in Table A1, and the analytical evaluation is provided in Table 4. The most substantial negative difference occurred in May, with a mobility decrease of 57.6%. Subsequently, we investigated the male and female groups in more depth. Table 5 provides a detailed comparison of average mobility between men and women.

### 3.4. Impact of City Size on Elderly Mobility During the Pandemic

Government-enforced measures were implemented nationwide. However, our focus was on determining whether these measures exerted a consistent impact on individuals who resided in urban or rural environments. To address this inquiry, we conducted a comparative analysis contrasting pre-COVID-19 mobility data with data from during the COVID-19 period, stratified across various categories of cities. More precisely, we classified a city as ‘big’ if its population exceeded 10,000 inhabitants, and as ‘small’ if it had fewer than 10,000 inhabitants.

Within this framework, hypotheses H05, H06, and H07 were formulated, and subsequent *p*-values were calculated utilizing paired t-tests for each of these hypotheses. When comparing the mobility data of individuals residing in big cities with those in small cities during both the pre-COVID-19 and COVID-19 periods, the observed variations in mobility were found to be consistent. In big cities, mobility decreased from 17.81 km per month to 9.43 km, while in small cities, it declined from 16.74 km to 11.08 km. As a result, we can infer that the city size did not exert a discernible influence on changes in mobility levels.

### 3.5. Mobility Variations by Type of Residence

We conducted a comparative analysis contrasting pre-COVID-19 data with data from during the COVID-19 period, specifically examining individuals with various types of living arrangements. Our research formulated hypotheses H08 and H09 for this purpose. Mobility data were available for 152 participants who had provided information about their type of residence.

Comparing the mobility patterns of participants living in flats or houses, we did not identify statistically significant differences. The mobility values for both groups are detailed in Table A2.

### 3.6. Age-Related Mobility Trends During COVID-19

We conducted a comparative analysis by contrasting pre-COVID-19 mobility data with data from during the COVID-19 period, with a specific focus on different age groups. Four distinct and sizable age groups were established for this analysis, and hypothesis H10 was formulated. Notably, the second (aged 80 to 85 years) and fourth age groups (aged more than 90 years) emerged as the most prominent. Table A3 presents the corresponding *p*-values for each age group.

Our findings indicate a statistically significant difference in mobility levels between the pre-COVID-19 and COVID-19 periods. Remarkably, the most active age group was the 80 to 85 years category, which exhibited an average mobility of 24.4 km during the pre-COVID period. However, in the period during COVID-19, this value decreased to 15,35 km, although it still exceeded the mobility levels of other age groups during the pre-COVID-19 period. Detailed results can be found in Table A2.

### 3.7. Relationship Between BMI (Body Mass Index) and Mobility

We aimed to investigate the possible impact of body mass index (BMI) on mobility within our study group. To achieve this, we analyzed the correlation between BMI and the distance traveled (in kilometers). This analysis was guided by hypotheses H11 and H12. The resulting correlation coefficients are detailed in Table A4. Results of the Pearson correlation indicated a very small non-significant negative relationship between BMI and mobility in the pre-COVID-19 period (r = −0.0646, *p* = 0.345). In contrast, the correlation between BMI and mobility during the COVID-19 period indicated a very small non-significant positive relationship (r = 0.0367, *p* = 0.591). Our analysis indicated no statistically significant correlation between BMI and mobility during either the pre-COVID-19 or COVID-19 periods.

## 4. Discussion

### 4.1. Telecare SOS Alarms

This study provides valuable insights into the impact of the COVID-19 pandemic on elderly mobility and safety in the Czech Republic. The relatively stable number of SOS alarms between the pre-COVID-19 period (2090 cases) and during COVID-19 (2019 cases) suggests that the overall need for assistance did not significantly change. However, the slight reduction in alarms triggered during the COVID-19 period may indicate that elderly individuals were more cautious or limited in their movement, potentially due to fear of infection or restrictions imposed by government regulations.

Notably, only 16.6% of all triggered SOS alarms required further action by the monitoring center, raising important questions regarding the utility and responsiveness of these alarms. The high proportion of alarms that did not necessitate intervention suggests a potential need for enhanced training in use of SOS devices or a refinement of the system in order to reduce false alarms and improve efficiency. This observation aligns with findings from previous studies on telemonitoring and emergency response systems, where a significant share of alarms did not indicate critical incidents but rather user errors or non-emergencies.

The observed reduction in the proportion of SOS alarms triggered outside the home (from 34% pre-COVID-19 to 29% during COVID-19) provides a clear indication of the pandemic’s impact on elderly mobility. This decrease was consistent with anticipated outcomes, as mobility restrictions, heightened fear of infection, and government guidelines were likely to have encouraged older adults to remain in their homes. Nevertheless, the fact that 29% of alarms continued to be activated outside the home during the pandemic suggests that some elderly individuals engaged in outdoor activities despite the restrictions, potentially due to essential needs or insufficient social support for tasks such as shopping and healthcare.

### 4.2. Mobility

The next objective of this study was to quantify the impact of COVID-19-related restrictions on elderly mobility (measured in kilometers per month) after March 2020. The results derived from objective measurements of mobility indicated an overall decline in mobility. The observed decrease in mobility, at 43.9%, was less substantial than suggested by the subjective survey data [22].A survey focusing on urban mobility found a decrease in trip frequency exceeding 50% [23], a trend similarly supported by Australian findings [24]. In contrast, our results demonstrated a reduction of 41.65%. A limitation of questionnaire-based surveys is that they may capture a general decline in elderly mobility but lack the precision to quantify the extent of this decrease [25].

A related study using GPS coordinates to calculate travel distances of residents in Osaka, Japan [26] reported that average weekly travel distances in 2020 were reduced by half compared with 2019. Although that study did not specifically target elderly individuals, its findings support our observations regarding mobility decline, though our results are specific to the elderly population. Comparative analysis with other studies is challenging, as our study examined two distinct periods separated by one year. Most surveys that have assessed mobility trends before and during the COVID-19 pandemic have tended to provide a generalized overview. Furthermore, studies employing GPS tracking have rarely focused specifically on elderly populations. Nevertheless, our findings of decreased mobility among elderly participants (216 individuals with an average age of 84 years) during the initial year of the pandemic are consistent with other studies [22,23,24,25,26,27], with a 44.7% decrease and an overall reduction of 41.7% in the study cohort during the COVID-19 period compared with pre-COVID-19. Restricted access to healthcare and shopping facilities during the pandemic probably influenced elderly mobility, either decreasing it due to fewer accessible locations or increasing it as individuals needed to manage essential tasks independently.

Our analysis of the association between BMI and mobility (r = −0.064 in the pre-COVID-19 period and r = 0.036 during the COVID-19 period) aligns with a study conducted in 2022 that reported minimal correlation between BMI and physical activity (r = −0.08 pre-COVID-19; r = 0.07 during COVID-19). This lack of substantial correlation suggests that BMI may not have a significant negative impact on mobility, indicating that excess weight might not necessarily be a barrier to mobility for elderly individuals.

Overall, our findings demonstrate a general decline in elderly mobility. Compared with previous studies using subjective data, our results show a smaller decline. However, 21 participants (9.72%) showed a 60.53% increase in travel distance during the pandemic, similar to findings from a UK-based study [28]. This increase may be attributed to the closure of home care services, which led seniors to manage daily activities independently. Factors such as housing type, BMI, age, and gender did not significantly impact the overall decline in mobility. Further analysis of the 9.72% of participants who showed an increase in mobility during the pandemic may suggest possible factors such as diminished caregiving support and an increased need to independently manage daily tasks. This subgroup warrants further investigation to understand the underlying motivators for increased mobility under restricted conditions.

The observed decline in mobility in December 2019 is likely to have been attributable to seasonal factors rather than COVID-19 concerns, as awareness of the pandemic was largely confined to China at that time. The marked decrease in mobility observed in April and subsequent months aligned with the onset of COVID-19 restrictions in the Czech Republic. The most substantial reductions in mobility observed for May, September, October, January, and February probably reflect peak COVID-19 periods, during which stricter lockdown measures and heightened caution were prevalent. The overall pattern of average monthly distances is illustrated in Figure 3. Notably, the first three confirmed COVID-19 cases in the Czech Republic were reported on 1 March 2020 [29]. Subsequently, the government declared a COVID-19 outbreak on 12 March 2020, as part of its renewed response to health threat of SARS-CoV-2 [30]. Shortly thereafter, retail sales and services were limited to essentials, and movement restrictions and mandatory mask mandates were enforced, prompting families to adopt protective measures, including isolating elderly members. Given the evolving regulatory environment, future research should expand this analysis with larger datasets to explore daily mobility patterns further. Specific measures, such as designated shopping hours for seniors, may have uniquely impacted this demographic’s mobility patterns.

### 4.3. Study Limitations

This study has several limitations that should be considered when interpreting its findings. The use of GPS tracker data, while providing objective mobility measurements, was not without errors. Erroneous data caused by reflections or inaccuracies in coordinates were addressed using filters, but residual inaccuracies may have persisted. Additionally, the retrospective design of the study may have introduced potential biases in data collection and analysis and thereby influenced the results.

Factors such as seasonal weather changes and pre-existing health conditions are likely to have influenced mobility levels independently of COVID-19 restrictions and were not accounted for in the analysis. Questionnaire-based surveys used for comparison also present limitations, as they capture general mobility trends but lack the precision to quantify mobility changes accurately.

The current results underscore the enhanced granularity and predictive value of studies utilizing objective measurements of mobility, particularly those derived from tracking devices. It is essential to corroborate conclusions from questionnaire-based surveys with precise objective measurements wherever possible. The general trend indicates a decline in mobility among 195 of our study participants, yet the average age of those with reduced mobility was nearly identical to that of participants with increased mobility. These findings suggest that factors beyond chronological age, such as mental state or genetic predisposition, may have played more influential roles. Additionally, the lack of significant differences in mobility decline between individuals living in houses versus apartments suggests that living conditions may not have substantially impacted mobility.

Variability in daily travel distances was observed, partly due to missing data for certain days. While days without recorded data were excluded to avoid skewing results, this approach may still have limited the robustness of the findings. Future studies could benefit from incorporating more precise wearable technology to improve data accuracy and consistency.

### 4.4. Elderly Mobility: Patterns, Challenges, and Strategies for Improvement

Elderly mobility exhibits distinct characteristics, appearing less influenced by living arrangements. Our analysis did not reveal significant differences in mobility decline between those living in houses versus apartments. Elderly individuals are likely to spend a considerable portion of time at home, suggesting that having a garden does not demonstrably enhance mobility. Conversely, elderly apartment residents may have increased opportunities for outdoor activities such as walks in nearby parks. Although this is plausible given the prevalence of apartments in urban areas, our findings do not support a definitive effect on mobility levels. Contrary to findings [18] in other countries, city size and residence type did not significantly impact mobility. This may reflect unique aspects of the Czech Republic, such as urban planning with accessible green spaces and the uniform implementation of quarantine measures across cities of different sizes.

Some participants showed substantial variability in daily travel distances. Variability in these distances can be attributed to the irregular patterns of activity among elderly individuals, who may spend some days at home while going out on others for essential activities like grocery shopping or medical appointments. As a result, promoting regular outdoor mobility becomes essential for this demographic. Maintaining a consistent minimum level of daily physical activity is vital, and supporting mobility is a key strategy. This may involve initiatives to improve elderly mobility within urban environments. Irregular physical activity heightens the risk of injuries and health complications in elderly populations, emphasizing the importance of consistent mobility practices.

Strategies to maintain regular mobility, such as safe designated outdoor spaces, community-led walking programs, and regular support check-ins, may provide critical support for elderly populations during periods of restriction. In cooperation with providers of tertiary social services, the authors of this article are developing new methods to support mobility in the elderly. Games represent an interesting method. Telemedicine could play a vital role in mitigating mobility needs during health crises, providing essential healthcare access without requiring physical travel. Further research could assess telemedicine’s effectiveness in maintaining elderly health outcomes during periods of restriction.

The current findings highlight the need for targeted interventions to support elderly mobility during public health emergencies, particularly in rural areas where access to services may be limited. Telemedicine services can provide essential healthcare access without requiring physical travel, reducing the burden on elderly individuals who may face challenges reaching medical facilities. Additionally, implementing outdoor mobility programs, such as community walking groups or designated safe outdoor spaces, can encourage physical activity and social engagement while maintaining adherence to public health guidelines. With a specific focus on rural settings, these tailored can mitigate the impact of restricted mobility during emergencies and promote overall well-being among elderly populations.

While this study provides foundational insights into elderly mobility patterns during the COVID-19 pandemic, further research is necessary to examine long-term effects and potential interventions. These findings underscore the need for door-to-door delivery services for essential items, such as medications and groceries, to maintain elderly autonomy and reduce exposure during future public health crises. Future studies could explore strategies to bolster mobility resilience among older adults, including targeted physical activity programs, digital engagement solutions, and support systems designed to reduce isolation during public health crises. Additionally, expanding the research to track trends in post-pandemic mobility may reveal whether these reductions in mobility have had enduring impacts on health outcomes for elderly populations. There will also be interest in incorporating wearable devices with higher accuracy to minimize data inconsistencies and increase the robustness of mobility measurements.

## 5. Conclusions

This study provides valuable insights into the impact of COVID-19 restrictions on elderly mobility and safety, highlighting both the resilience of this vulnerable population and the challenges they have faced. The findings demonstrate a significant decline in mobility, with an overall reduction of 43.9% during the pandemic. While this decrease is to be expected given the restrictions, these circumstances underscore the necessity of targeted interventions to mitigate such impacts during future public health crises.

The analysis of SOS alarms revealed that while most alarms did not require intervention, the high proportion of false activations suggests a need for system refinements and user training. These improvements could enhance the efficiency and utility of emergency care systems for elderly individuals. Furthermore, the observed variability in mobility patterns, particularly among those who have shown increased activity, points to the importance of understanding individual needs and circumstances.

Strategies to support the mobility of elderly people during emergencies should include telemedicine services, community-led outdoor programs, and policies such as door-to-door delivery of essential goods. These measures can help maintain physical activity, reduce isolation, and ensure access to critical resources.

Future research should expand on these findings by exploring the long-term effects of mobility reductions and investigating tailored interventions for specific subgroups, including those in rural or urban settings. Understanding the nuanced needs of elderly populations will be essential for enhancing their resilience and autonomy in the face of future health emergencies.

## Figures and Tables

**Table 1:** Demographic characteristics of the 216 participants, including age distribution, gender, BMI categories, residence type, and other relevant attributes. Data are presented as frequencies (n) and percentages (%), providing a comprehensive overview of the study population.

	Number/Mean ± SD	n (%)
**Gender**		
Male	48	22.22
Female	168	77.78
**Physical characteristics**		
Weight ± SD	74.52 ± 13.77 kg	
Height ± SD	164.62 ± 8.45 cm	
BMI ± SD	26.48 ± 3.78	
**Mobility**		
No problems	72	33.49
Small problems	64	29.77
With help	50	23.26
Wheelchair	2	0.93
Not defined	27	12.56
**House**		
In flat	106	49.07
In house	46	21.30
With family	38	17.59
Not defined	26	12.04
**City size**		
Less than 100 k (small city)	101	46.76
Over than 100 k (big city)	115	53.24
**Age group**		
<80	43	14.63
80–85	71	24.15
86–90	59	20.07
>90	43	41.16
**Age**		
Max age	102	
Average age ± SD	84.01 ± 7.3	

**Table 2:** Number of emergency care service SOS alarms triggered during the pre-COVID-19 period (March 2019–February 2020) and during COVID (March 2020–February 2021). Data include total alarms, alarms requiring intervention, and their respective proportions.

	Number	Percentage	Pre-COVID	During-COVID
**Alarms**				
SOS button pushed	4109		2090	2019
Fall detection	300		137	163
**Method of resolution**				
Resolved with assistance	732	16.60%	405	327
Test	508	11.52%	223	285
Pressed by mistake	2438	55.30%	1233	1205
Other	731	16.58%	366	365
**Alarms with fall**				
Fall	124		47	76
Fall assistance required	41		17	24
**Alarm location**				
Occurrence at home	2076	47%	1036	1040
Occurrence outside the home	1395	32%	757	638
Could not be determined	938	21%	434	504

**Table 3:** Distribution of participants’ changes in mobility (decreased or increased) between the pre-COVID-19 and COVID-19 periods. Values are presented as frequencies (n) and percentages (%).

Status	Number	Percentage	Age	Mobility Difference
Mobility increased	21	9.72%	84	60.53%
Mobility declined	195	90.28%	85	−56.94%

**Table 4:** Comparison of average monthly mobility levels (in kilometers) between the pre-COVID-19 and COVID-19 periods. Data are presented as means ± SD, with statistical significance indicated where applicable.

Month	Average Mobility in km in Pre-COVID-19 Group	Average Mobility in km in During-COVID-19 Group	Difference in %
March	19.39	11.25	−42.0
April	20.05	11.71	−41.6
May	22.33	9.47	−57.6
June	20.82	12.12	−41.8
July	19.07	14.29	−25.1
August	16.26	11.59	−28.7
September	18.95	9.23	−51.3
October	16.09	7.73	−51.9
November	15.11	11.56	−23.5
December	12.40	9.91	−20.1
January	14.10	6.58	−53.4
February	13.23	6.37	−51.8

**Table 5:** Comparison of average monthly mobility levels (in kilometers) between men (M) and women (F) during the pre-COVID-19 and COVID-19. Data are presented as means ± SD, with statistical significance indicated for gender-based differences.

Month	Pre-COVID		During-COVID		Difference in %	
	M	F	M	F	M	F
January	12.05	21.37	12.12	10.87	0.6	−49.1
February	14.01	21.65	14.37	10.47	2.6	−51.6
March	13.41	24.88	6.30	10.27	−53.0	−58.7
April	16.68	21.75	9.64	12.82	−42.2	−41.0
May	12.10	21.06	9.03	15.79	−25.4	−25.0
June	11.45	17.43	8.14	12.51	−28.9	−28.3
July	12.03	20.92	7.92	9.60	−34.1	−54.1
August	16.06	15.81	5.51	8.27	−65.7	−47.7
September	18.65	14.01	8.24	12.37	−55.8	−11.7
October	18.66	10.46	7.28	10.60	−61.0	1.3
November	19.67	12.43	5.11	6.88	−74.0	−44.6
December	20.17	11.01	8.64	5.31	−57.2	−51.8

## Data Availability

GPS data and client data are available upon request. To request data, readers should contact the corresponding author: V.J. Other input data are public, and the reader can use the link in the references section. Details regarding analytical methods, including Python scripts, can be requested from the corresponding author: M.M. The data are not publicly available due to their large size.

## Appendix A

### Established Questions

For the evaluation, we posed the following questions. Table A3 displays the *p*-values associated with these hypotheses.

- H01: Overall participant mobility remained unchanged between the pre-COVID-19 and COVID-19 periods.
- H02: Male mobility did not differ between the pre-COVID-19 and COVID-19 periods.
- H03: Female mobility remained consistent between the pre-COVID-19 and COVID-19 periods.
- H04: Participant mobility was the same each month before and during COVID-19.
- H05: Mobility in large cities did not change between the pre-COVID-19 and COVID-19 periods.
- H06: The mobility of participants in small cities was similar to those in large cities during the pre-COVID-19 period.
- H07: The mobility of participants in small cities was similar to those in large cities during the COVID-19 period.
- H08: Pre-COVID-19 mobility was the same for participants living in flats or houses.
- H09: During COVID-19, mobility was the same for participants living in flats or houses.
- H10: Mobility did not change between the pre-COVID-19 and COVID-19 periods within any age group.
- H11: There was a correlation between BMI and average mobility in the pre-COVID-19 period.
- H12: There was a correlation between BMI and average mobility during the COVID-19 period.

**Table A1:** Monthly statistical comparison for Hypothesis H04, evaluating differences in participant mobility between the pre-COVID-19 and COVID-19 periods. For each month, *p*-values, results of normality tests for both groups, and the type of statistical test used (Wilcoxon) are presented.

Month	*p*-Value	Normality Test Result Pre-COVID Group	Normality Test Result During-COVID Group	Test Type
March	<0.001	No	No	Wilcox
April	<0.001	No	No	Wilcox
May	<0.001	No	No	Wilcox
June	<0.001	No	No	Wilcox
July	<0.001	No	No	Wilcox
August	<0.001	No	No	Wilcox
September	<0.001	No	No	Wilcox
October	<0.001	No	No	Wilcox
November	<0.001	No	No	Wilcox
December	<0.001	No	No	Wilcox
January	<0.001	No	No	Wilcox
February	0.001	No	No	Wilcox

**Table A2:** Statistical parameters, including mean, standard deviation (SD), median, and sample count, for hypotheses related to mobility before and during the COVID-19 pandemic. Data are presented in kilometers and categorized by various factors, including hypothesis-specific comparisons, city size, residence type, and age group.

Hypothesis		Mean		Std		Median		Sample Count
		Pre-COVID-19	During COVID-19	Pre-COVID-19	During COVID-19	Pre-COVID-19	During COVID-19	
H01		17.31	10.20	52.25	38.06	4.42	1.55	216
H02		15.46	8.56	33.47	18.19	6.67	2.11	48
H03		17.84	10.67	56.55	42.08	4.17	1.49	168
H05		17.81	9.43	52.73	39.57	4.71	1.63	115
H06 + H07	Big city	17.81	9.43	52.73	39.57	4.71	1.63	115
	Small city	16.74	11.08	51.95	36.44	4.15	1.42	101
H08 + H09	Flat	26.17	15.36	69.98	43.26	5.18	2.13	46
	House	19.57	11.69	57.57	45.55	4.53	1.5	106
H10 (<80)		8.36	4.82	12.94	12.9	3.11	1.02	43
H10 (80–85)		24.4	15.35	67.45	54.82	6.26	2.34	71
H10 (86–90)		23.72	13.36	64.89	38.75	4.64	1.86	59
H10 (>90)		5.76	2.74	6.89	4.58	4.22	1.23	43

**Table A3:** *p*-values for statistical tests conducted to evaluate each hypothesis, comparing mobility levels and related factors between the pre-COVID-19 and COVID-19 periods.

Hypothesis	*p*-Value	Test Type
H01	<0.001	Wilcox
H02	<0.001	Wilcox
H03	<0.001	Wilcox
H05	<0.001	Wilcox
H06	0.208	Wilcox
H07	0.744	Wilcox
H08	0.281	Wilcox
H09	0.286	Wilcox
H10 (<80)	<0.001	Wilcox
H10 (80–85)	<0.001	Wilcox
H10 (86–90)	<0.001	Wilcox
H10 (>90)	<0.001	Wilcox

This table shows the statistical values for each hypothesis, with values provided for each group comparison.

**Table A4:** Correlation analysis between BMI and mobility levels, with H11 representing the pre-COVID-19 period and H12 representing the period during COVID-19. Data include Pearson correlation coefficients (r-values) and associated *p*-values for statistical significance.

Hypothesis	R-Value	*p*-Value
H11	−0.064	0.345
H12	0.036	0.591

## Appendix B

### Appendix B.1. Resource Mobility Data Package

File Content (JSON)

- personID {number}
- timestamp {yyyy-mm-ddThh:mm.s}
- geoType {type}
- accuracy {number}
- latitude {number}
- longitude {number}

In our study, GPS coordinates were used to calculate the total distance covered by the senior participants. To ensure data quality, a filter was applied to eliminate inaccuracies and artefacts. The exact description of the filtration is in Section 2.3.

### Appendix B.2. Client Information File

JSON format

- Client ID {number}
- Gender {text}
- Year of birth {number}
- Postcode of residence {number}
- Mobility Status {normal, low, with help, wheelchair}
- Housing Status {apartment, house}
- Height {number}
- Weight {number}

### Appendix B.3. City Population Numbers

XLSX format

- District code {number}
- City code {number}
- Name of municipality {text}
- Population total, males, females {number}
- Average age total, males, females {number}

Document published by the Czech Statistics Office: Population of Municipalities in the Czech Republic [31].

### Appendix B.4. ZIP Code List

XLS format

- City name {text}
- ZIP code {number}
- Post name {text}
- District code {number}
- District name {text}

This document, published by the Czech Post Company, provides a list of ZIP codes for municipal sections and entire municipalities without subdivisions [32].

### Appendix B.5. Alarm List

XLS format

- Client ID {number}
- Alarm ID {number}
- Type resolve {text}
- GPS client home {number}
- GPS alarm {number}
- Timestamp {yyyy-mm-ddThh:mm.s}
